# A Low-Viscosity Copper-Based Deep Eutectic Solvent for Carbon Monoxide Elimination at the Return Corner of Coal Mining Faces

**DOI:** 10.3390/molecules31050772

**Published:** 2026-02-25

**Authors:** Tianyu Xin, Xihua Zhou, Yashengnan Sun, Gang Bai, Weiji Sun, Junguang Wang, Bing Liang, Jiaxu Jin

**Affiliations:** 1 School of Safety Science and Engineering, Liaoning Technical University, Huludao 125105, China; 2 School of Mechanics and Engineering, Liaoning Technical University, Fuxin 123000, China; 3 School of Architecture and Construction, Liaoning Technical University, Fuxin 123000, China

**Keywords:** goaf, return air corner, deep eutectic solvents (DESs), carbon monoxide (CO), CO elimination

## Abstract

To address the recurring issue of excessive carbon monoxide (CO) concentrations at the return corner of fully mechanized mining faces under goaf conditions, this study investigated the elimination of CO at ambient temperature and pressure using deep eutectic solvents (DESs). CO, a colorless, odorless, and highly toxic gas, is notoriously difficult to remove under conventional conditions. A series of DESs were prepared and screened, revealing that the ethanol-modified system [Emim]Cl-CuCl-1.0E exhibited optimal CO elimination performance under conditions of 298.15 K and atmospheric pressure. Further investigations measured the viscosity-temperature relationship and thermal stability of this system while systematically examining the effects of temperature, CO contact time, and storage duration on its elimination efficiency. Analysis by FTIR and Raman spectroscopy indicated that Cu(I) ions play a crucial role in the CO absorption process. The introduction of ethanol significantly enhanced the activity of the Cu(I) ions, thereby effectively improving the CO elimination capacity of the system. This study proposes a novel potential method for managing CO in goaf areas and provides an experimental foundation for the application of DESs in the field of gas purification.

## 1. Introduction

In coal mining operations, shallow and contiguous goaf areas are significantly affected by residual coal pillars and mining-induced fractures in the overlying strata. These factors create complex ventilation leakage pathways connecting the surface, underground workings, and between layers, providing an ample oxygen supply for the residual coal in the current seam’s goaf and the remnant pillars in the overlying goaf [1,2]. This, in turn, influences the production and migration of carbon monoxide (CO) within the goaf and often leads to CO concentrations exceeding safety limits at the return corner of the working face [3]. This phenomenon poses significant challenges for CO control and the early prediction and warning of coal spontaneous combustion at the return corner. Current CO elimination methods primarily include catalytic oxidation [4,5,6,7,8,9] and chemical adsorption [10,11,12,13]. These techniques are mainly applied in fields such as automotive and industrial exhaust treatment, where they target CO before it is emitted into the atmosphere. However, there is a lack of effective methods for eliminating CO that is already present in the ambient air.

Deep Eutectic Solvents (DESs) [14,15,16,17,18,19,20,21] represent one of the most advanced solvents in the field of separation, consisting of hydrogen bond donors and acceptors. The first example of a DES was reported by Abbott et al. [22], demonstrating its formation through the reaction of choline chloride with a range of carboxylic acids and its straightforward preparation. Notably, DESs not only retain many advantages of Ionic Liquids (ILs) but also overcome certain limitations of ILs, such as toxicity and biocompatibility concerns [23,24,25]. To date, researchers have developed various DES systems for CO absorption. Tao et al. [26] discovered that the ternary DES [BimH]C1-CuCl-1.0ZnCl_2_ exhibited a very high CO absorption capacity (0.075 mol/mol, 1.0 bar), superior to all previously reported absorbents, even at elevated temperatures (353.2 K). Cui et al. [27] demonstrated that the presence of Cu(I) is crucial for CO capture and that OH groups and ethylene glycol (EG) can enhance the activity of Cu(I). Zhu et al. [28] found that the DES [Emim]Cl-CuCl-ZnCl_2_-EG achieved a record-breaking CO absorption capacity (0.934 mol/mol, nearly equimolar CO capture) at 1.0 bar and 298.2 K. Spectroscopic characterization indicated that ZnCl_2_ and EG weaken the Cu-Cl bond, causing a significant redshift, which facilitates stronger interaction between Cu^+^ species and a greater number of CO molecules, thereby enhancing the CO capacity. Gong et al. [29] developed a low-viscosity bimetallic DES, [Emim]Cl-CuCl-1.0FeCl_3_, with high CO absorption capacity, reaching near-equimolar capture (0.9 mol/mol) at 298.2 K and 100 kPa. However, most existing studies have been conducted under high-pressure conditions, focusing primarily on CO absorption capacity and selectivity. Their applicability in the atmospheric pressure environment typical of a return air corner remains unclear.

Herein, a variety of DESs were prepared. Through performance evaluation experiments, the ethanol-modified system [Emim]Cl-CuCl-1.0E was identified as the optimal candidate for CO elimination under conditions of 298.15 K and atmospheric pressure. Subsequently, its physicochemical properties—including the viscosity-temperature relationship and thermal stability—were comprehensively investigated. The CO elimination performance was systematically evaluated under varying ambient temperatures, elimination times, and sealed storage durations. Furthermore, the CO adsorption sites and the underlying interaction mechanism were elucidated using Fourier-transform infrared (FTIR) and Raman spectroscopy.

## 2. Results and Discussion

### 2.1. CO Capture Measurements

**Figure 1:**
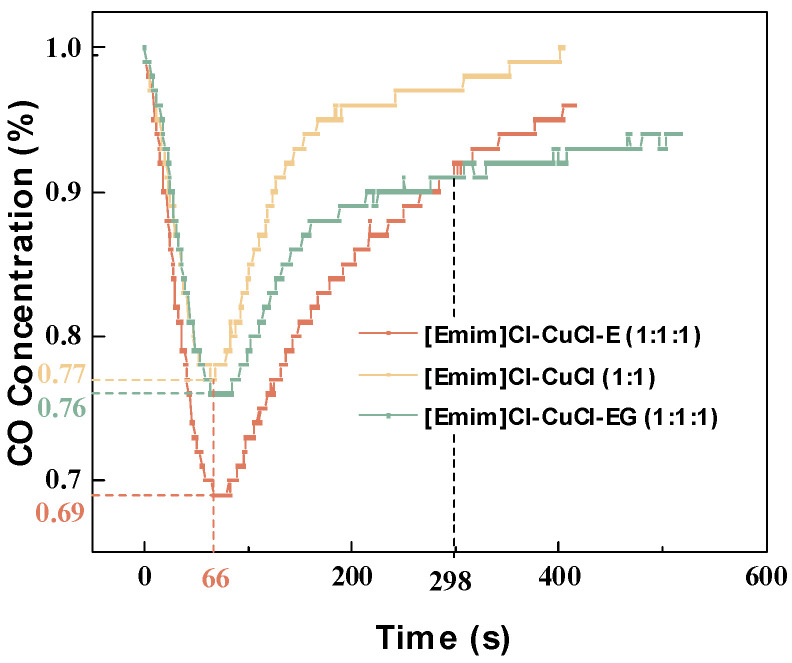
Real-time CO concentration profiles of DESs with different modification methods during testing.

To evaluate the impact of ethanol on the CO absorption performance of copper-based DESs, tests were conducted under conditions of 298.15 K and atmospheric pressure. The performance was compared with that of the most commonly used modifier, ethylene glycol, as shown in Figure 1, [Emim]Cl-CuCl-1.0E exhibited the best CO absorption performance. This system reduced the initial CO concentration from 1% to 0.69% during the contact period, achieving CO elimination efficiencies of 20.9%, 17.8%, and 14.9% at 200 s, 300 s, and 400 s, respectively. In comparison, the elimination efficiencies for [Emim]Cl-CuCl-1.0EG and [Emim]Cl-CuCl at the same time points were 15.5%, 13.6%, and 12.2% and 15.45%, 9.0%, and 7.1%, respectively. These results demonstrate that both ethanol and EG aid in activating the Cu(I) sites, thereby improving the CO absorption capacity of the Cu(I)-based DESs, with ethanol providing a superior promotional effect compared to EG. It is noteworthy that at 298 s, the CO absorption capacity of the EG-modified DES began to exceed that of the ethanol-modified system. This is likely attributable to the faster occupation and oxidation of the Cu(I) sites in the ethanol-modified DES.

**Figure 2:**
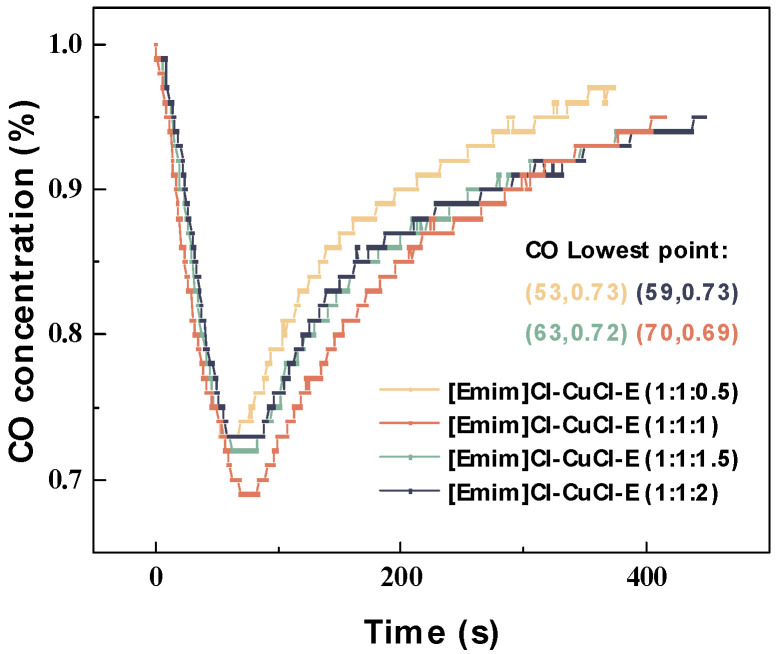
Real-time CO concentration profiles of [Emim]Cl-CuCl-1.0E with different ethanol molar ratios during testing.

Furthermore, the influence of ethanol dosage on the CO absorption performance of the DESs was investigated under identical conditions; the results are shown in Figure 2. It can be observed that [Emim]Cl-CuCl-1.0E possessed the highest CO absorption performance, yielding a minimum CO concentration of 0.69%. This value is slightly lower than the corresponding values of 0.73%, 0.72%, and 0.73% for [Emim]Cl-CuCl-0.5E, [Emim]Cl-CuCl-1.5E, and [Emim]Cl-CuCl-2.0E, respectively. This suggests that while the addition of ethanol helps enhance the activity of Cu(I) ions, an excessive amount of ethanol leads to a decrease in the concentration of Cu(I) ions in the solution, consequently impairing the absorption performance.

### 2.2. Physical Properties

**Figure 3:**
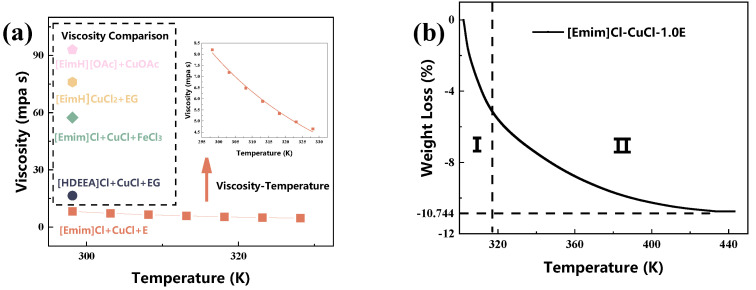
Viscosity of [Emim]Cl-CuCl-1.0E as a function of temperature and its comparison with viscosities of other elimination agents reported in the literature at 298.15 K (**a**). Thermogravimetric (TG) analysis curve of [Emim]Cl-CuCl-1.0E (**b**).

Viscosity is a critical property of DESs, significantly influencing their CO absorption capacity. As temperature markedly affects the viscosity of DESs, the viscosity of [Emim]Cl-CuCl-1.0E was measured within the temperature range of 298.15 K to 328.15 K. For comparison, the viscosities of several other elimination agents at 298.15 K were also selected from references [27,29,30,31]; the results are presented in Figure 3a. The results indicate that the viscosity of [Emim]Cl-CuCl-1.0E is 8.2 mPa·s at 298.15 K, which is significantly lower than that of the other elimination agents. This suggests lower mass transfer resistance during the CO absorption process, a characteristic that has been demonstrated to be favorable for CO absorption. Furthermore, the viscosity was observed to decrease with increasing temperature, a relationship that can be described by Equation (2) (Fitting parameters: $u_{0}$ = 0.01387, $E_{a}$ = 15,778.11775).

Due to the addition of ethanol, the thermal stability of [Emim]Cl-CuCl-1.0E was further investigated, with the results shown in Figure 3b. It can be clearly observed that the thermogravimetric (TG) curve for [Emim]Cl-CuCl-1.0E is concave. The weight loss exhibited a pattern of being rapid initially and then slowing down. This phenomenon can be attributed to the different states of ethanol within the system: the initial rapid weight loss corresponds to the volatilization of excess or free ethanol. As this excess ethanol evaporates, the subsequent, slower weight loss stems from the ethanol molecules that are involved in hydrogen bonding with the system. The volatilization of this bound ethanol requires overcoming a higher energy barrier, resulting in a significantly reduced rate.

(1) $$u=u_{0}\exp\left(\frac{E_{a}}{RT}\right)$$

Here, u is the dynamic viscosity/mPa·s, $u_{0}$ is the pre-exponential factor, $E_{a}$ is the activation energy/kJ·mol^−1^, R is the universal gas constant (8.314 J·mol^−1^·K^−1^), and T is the absolute temperature/K.

### 2.3. Analysis of Influencing Factors

The CO elimination performance of [Emim]Cl-CuCl-1.0E was evaluated under varying conditions of ambient temperature, contact time with CO, and sealed storage time.

**Figure 4:**
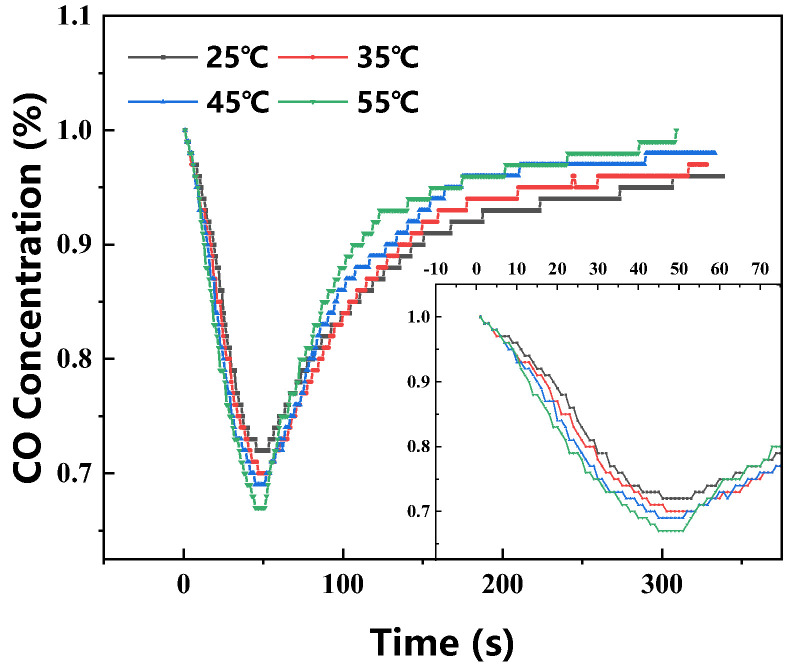
Real-time CO concentration profiles of [Emim]Cl-CuCl-1.0E at different temperatures during testing.

Figure 4 illustrates the influence of temperature on [Emim]Cl-CuCl-1.0E. The study found that temperature affects both the minimum CO concentration reached and the elimination efficiency during the CO elimination process. The minimum CO concentrations at 298.15 K, 308.15 K, 318.15 K, and 328.15 K were 0.72%, 0.70%, 0.69%, and 0.67%, respectively, showing a clear negative correlation. Elevated temperatures help reduce the mass transfer resistance of the solvent and enhance gas diffusion rates, thereby promoting the forward reaction. Regarding CO elimination efficiency, the efficiencies within 200 s and 300 s across the same temperature range were 14.9%, 15.2%, 14.4%, 13.7% and 11.9%, 11.7%, 10.6%, 9.9%, respectively. This indicates that increasing the temperature reduces the system’s saturated adsorption capacity, which aligns with previous research [32].

In the elimination experiments, the contact time between [Emim]Cl-CuCl-1.0E and the CO gas mixture significantly influenced CO elimination. To study this effect, quantitatively describing the contact time is essential. Within the solvent, the CO gas exists in the form of bubbles. Due to the complex motion behavior of bubbles in the liquid, this study employed video recording to quantitatively analyze the contact time of CO in [Emim]Cl-CuCl-1.0E.

**Figure 5:**
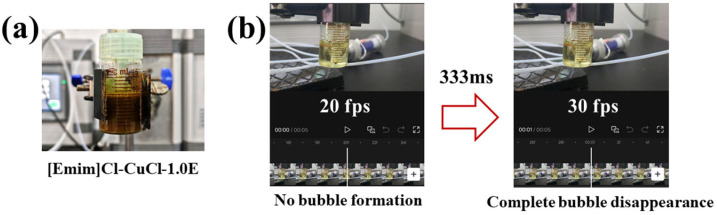
Substitution experiment using soybean oil to visualize bubble motion. Opaque [Emim]Cl-CuCl-1.0E sample (**a**). Quantitative analysis of CO elimination time in [Emim]Cl-CuCl-1.0E (**b**).

The [Emim]Cl-CuCl-1.0E sample is an opaque liquid, as shown in Figure 5a. To visualize the bubble motion trajectory, soybean oil with a similar viscosity (8.5 mPa s) was used as a substitute for the [Emim]Cl-CuCl-1.0E sample in the quantitative analysis experiment for CO elimination time [33]; the result is presented in Figure 5b. Calculations based on the video analysis indicate that the approximate residence time of a single bubble in the solvent is 333 milliseconds.

**Figure 6:**
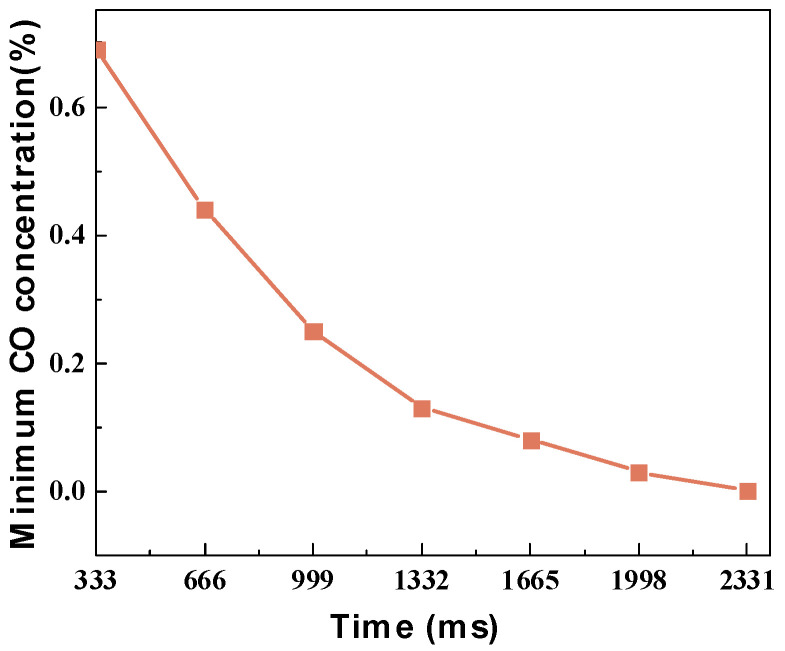
Effect of contact time on the minimum CO concentration during the elimination process using [Emim]Cl-CuCl-1.0E.

To further extend the contact time between CO and [Emim]Cl-CuCl-1.0E, the number of elimination bottles connected in series was increased. The results, shown in Figure 6, demonstrate that the minimum achievable CO concentration gradually decreased as the contact time increased. When the number of elimination bottles reached seven, CO was completely eliminated for the first time, corresponding to a total contact time of approximately 2331 milliseconds (about 2.3 s). This indicates that [Emim]Cl-CuCl-1.0E can achieve complete CO elimination within a short timeframe.

**Figure 7:**
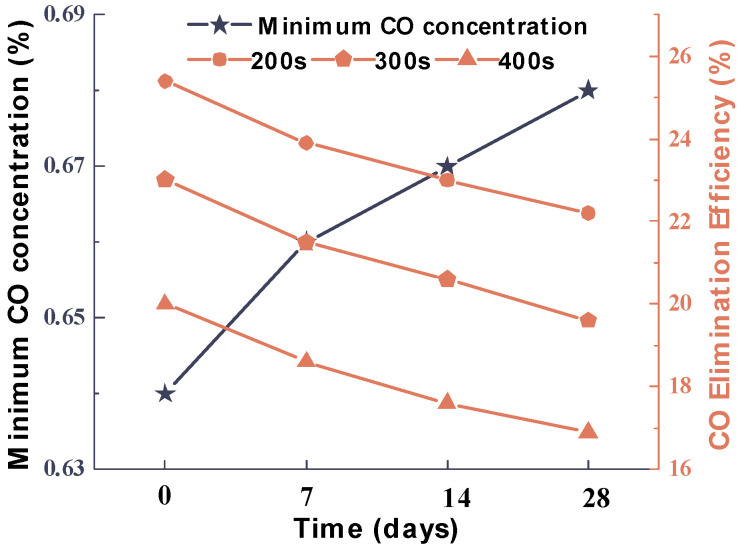
Effect of sealed storage time on the minimum CO concentration (left) and elimination efficiency (right) during the elimination process using [Emim]Cl-CuCl-1.0E.

To investigate the effect of storage time, [Emim]Cl-CuCl-1.0E was stored in a solvent bottle exposed to the ambient environment, and its CO elimination performance was evaluated. The results are presented in Figure 7. The CO elimination performance of the absorption system showed significant degradation with prolonged storage time. After 28 days of storage, the elimination efficiencies measured at 200 s, 300 s, and 400 s decreased from their initial values of 25.4%, 23.0%, and 20.0% to 22.2%, 19.6%, and 16.9%, respectively. Concurrently, the minimum achievable CO concentration increased from approximately 0.63% to 0.69%. These data collectively indicate that the CO elimination performance of [Emim]Cl-CuCl-1.0E gradually deteriorates with extended storage.

### 2.4. Activating Role of EGOH

**Figure 8:**
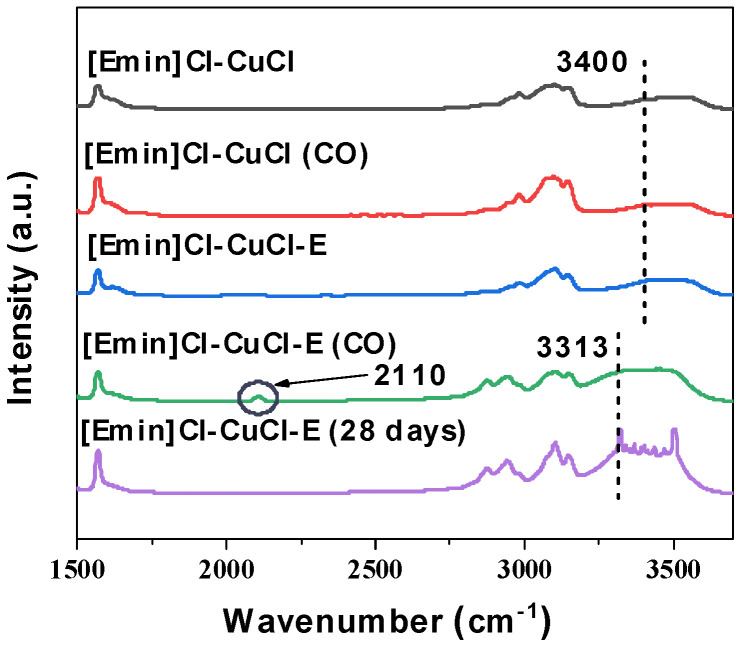
FTIR spectra of [Emim]Cl-CuCl-1.0E before and after CO elimination and after 28 days of sealed storage.

Fourier Transform Infrared (FTIR) spectroscopy was employed to characterize [Emim]Cl-CuCl and [Emim]Cl-CuCl-1.0E before and after CO absorption under conditions of 293.2 K and atmospheric pressure. The results revealed that, compared to the spectrum of the pristine sample in Figure 8, a new peak appeared at 2020 cm^−1^ in the spectrum of [Emim]Cl-CuCl-1.0E after CO adsorption. This indicates the formation of a stable Cu(CO)^+^ complex through the interaction between Cu(I) and CO within the system. In contrast, no significant changes were observed in the FTIR spectra of [Emim]Cl-CuCl before and after CO exposure across multiple experiments. This absence of spectral change is attributed to the minimal amount of CO absorbed by [Emim]Cl-CuCl within the same timeframe, which further confirms that ethanol effectively activates the Cu(I) ions in the system. Moreover, the absorption band corresponding to the CO stretching vibration was red-shifted from 2143 cm^−1^ (free state) to 2110 cm^−1^. This shift demonstrates that the addition of ethanol enhances the interaction between Cu(I) ions and CO, providing further evidence for its activating role.

**Figure 9:**
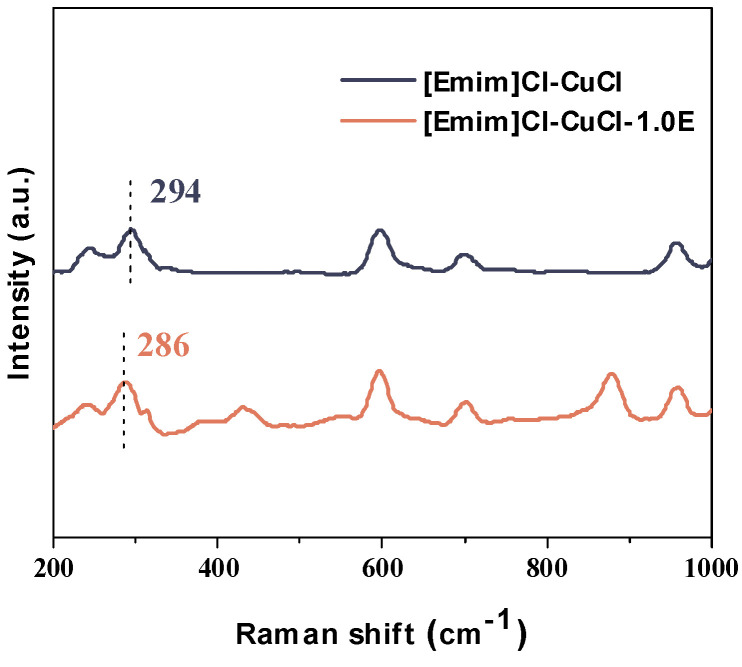
Raman spectra of [Emim]Cl-CuCl and [Emim]ClCuCl-1.0E.

Raman spectroscopy was further employed to elucidate the enhancing role of ethanol in the effective CO absorption by the [Emim]Cl-CuCl system, as presented in Figure 9. The characteristic peak for Cu-Cl vibrations in pristine [Emim]Cl-CuCl was observed at 294 cm^−1^. In contrast, the Cu-Cl band in the ethanol-modified [Emim]Cl-CuCl-1.0E exhibited a distinct red shift to 286 cm^−1^. This shift indicates a weakening of the Cu-Cl bond, which facilitates an increase in the coordination number of the Cu^+^ ion, thereby promoting its coordination with a greater number of ligand molecules. These findings are consistent with previous reports in the literature [28,29].

To investigate the underlying cause of performance degradation after prolonged storage, Fourier-transform infrared (FTIR) spectroscopy was performed on the [Emim]Cl-CuCl-1.0E sample after 28 days of storage. The results are presented in Figure 8. Compared to the fresh sample, the O-H stretching vibration band in the stored sample exhibited a red shift from 3400 cm^−1^ to 3313 cm^−1^, accompanied by a noticeable change in band shape. This suggests a reorganization or weakening of the hydrogen bond network within the solvent during storage, a phenomenon potentially linked to the ingress of atmospheric components such as moisture and oxygen. Notably, a characteristic band near 3313 cm^−1^ also appeared in the spectrum of the [Emim]Cl-CuCl-1.0E sample after CO adsorption ([Emim]Cl-CuCl-1.0E (CO)), indicating that exposure to the CO gas mixture likewise influences the hydrogen bonding structure of the system.

**Figure 10:**
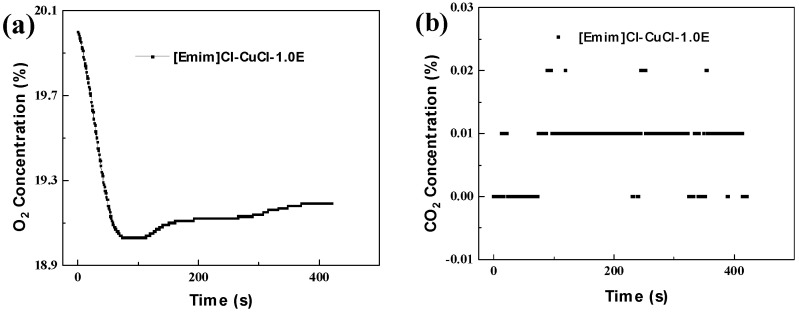
Real-time concentrations of O_2_ (**a**) and CO_2_ (**b**) during the testing of [Emim]Cl-CuCl-1.0E.

To clarify whether oxygen participates in the reaction process, the concentrations of O_2_ and CO_2_ in the CO gas mixture were measured before and after the experiments. The results are shown in Figure 10. Figure 10a reveals a decrease in O_2_ content of approximately 1% after the experiment, confirming that oxygen participates in reactions within the system. Conversely, Figure 10b shows that the amount of CO_2_ generated was minimal. Therefore, the consumption of oxygen is not attributable to its reaction with CO to form CO_2_ but is more likely due to reactions with other components in the system, which may negatively impact the CO elimination performance.

### 2.5. Recycle Test

**Figure 11:**
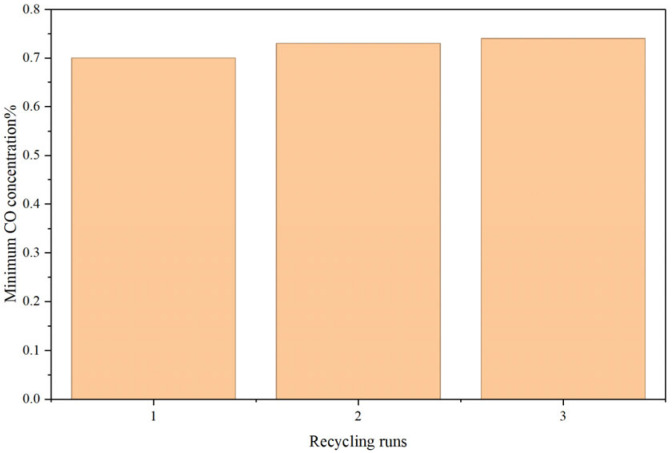
Recycle performance of [Emim]Cl-CuCl-1.0E for CO absorption.

To determine the reversibility of [Emim]Cl-CuCl-1.0E, three cycle tests were carried out after treating the used [Emim]Cl-CuCl-1.0E by multiple methods, including vacuum treatment. As can be seen from Figure 11, the CO adsorption capacity of the composite ionic liquid [Emim]Cl-CuCl-1.0E was well preserved to a certain extent, confirming that the CO adsorption process is reversible. However, the performance of the composite ionic liquid decreased slightly after repeated treatment under the current methods.

## 3. Materials and Methods

### 3.1. Materials

The following chemicals were used: 1-Ethyl-3-methylimidazolium chloride ([Emim]Cl, 98%), Copper(I) chloride (CuCl, 97%), Ethylene Glycol (EG, AR grade), Anhydrous Ethanol (EGOH, 99%), and a CO gas mixture (Standard gas, 1% CO, 20% O_2_, 79% N_2_).

### 3.2. DESs Preparation and Waste Treatment

The DESs used in this study were prepared via a heating and mixing method. Initially, [Emim]Cl and ethanol were mixed at molar ratios of 1:x (x = 0.5, 1.0, 1.5, 2.0) at room temperature, sealed, and stirred for 10 min. Subsequently, the [Emim]Cl-ethanol mixture was combined with CuCl at a molar ratio of 1:1. The sealed mixture was then heated in a water bath at 55 °C for one hour to obtain the ethanol-activated copper-based DESs, designated as [Emim]Cl-CuCl-xE. Using a similar procedure, ethylene glycol-activated copper-based DESs were prepared and named [Emim]Cl-CuCl-xEG. The non-activated copper-based DES was prepared and named [Emim]Cl-CuCl.

The viscosity of the solvents was measured in the temperature range of 298.15 K to 318.15 K using a Pinkevich capillary viscometer (Want Balance Instrument Co., Ltd., Changzhou, China). The CO concentration was analyzed using a Shenzhen Sentronictek CO analyzer (Shenzhen, China) (error: ±0.1%). The O_2_ concentration was analyzed using a Shenzhen Sentronictek O_2_ analyzer (error: ±0.1%). The CO_2_ concentration was analyzed using a Shenzhen Sentronictek CO_2_ analyzer (error: ±0.1%). Thermogravimetric analysis (TGA) was performed using an EVO2G-TG analyzer (Rigaku Corporation, Tokyo, Japan) under N_2_ atmosphere from room temperature to 443.15 K. The FTIR and Raman spectra of the solvents were obtained using a TENSOR 27 Fourier-transform infrared spectrometer (Bruker, Billerica, MA, USA) and a Horiba LabRAM HR Evolution Raman spectrometer (Horiba, Kyoto, Japan), respectively.

Upon completion of the experiments, the CO absorption solvent was uniformly collected, sealed, and entrusted to qualified professional institutions for centralized harmless disposal in accordance with laboratory regulations on hazardous chemicals and organic waste liquids. No waste liquid or gas was directly discharged during the experiments. The entire procedure complies with environmental safety and emission requirements, and no illegal discharge occurred.

### 3.3. DES Performance Evaluation

**Figure 12:**
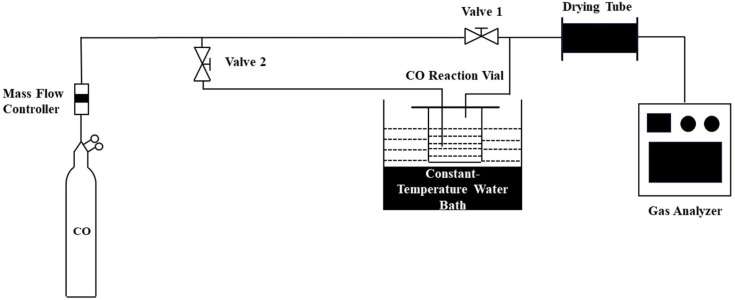
Schematic diagram of the CO elimination experimental setup.

The experimental setup used for evaluating the performance of the CO elimination agents is shown in Figure 12. In this work, the prepared copper-based DESs were placed into a 15 mL reaction vial made of borosilicate glass, with a fixed volume of 9 mL used for the CO elimination performance assessment. The experimental procedure was as follows: first, valve 1 was opened and valve 2 was closed until the CO reading stabilized. Subsequently, the copper-based DES was introduced into the CO reaction vial, then valve 1 was closed and valve 2 was opened to initiate the elimination experiment. The test conditions employed were a temperature of 298.15 K, atmospheric pressure, a gas composition of 1% CO, 20% O_2_, and 79% N_2_, and a gas flow rate of 40 mL/min. The reaction time was varied by connecting 1 to 7 reaction vials in series. The gas composition at the reactor inlet was controlled by mass flow controllers, while the composition at the outlet was analyzed in real-time by gas analyzers.

This study employed two metrics to evaluate the CO absorption performance of the DESs: the lowest CO concentration reached during the absorption process and the elimination efficiency. The CO elimination efficiency (*η*) is defined as the ratio of the amount of CO eliminated by the solvent to the total amount of CO introduced through the solvent layer within a given time period, calculated according to Equation (1):

(2) $$\eta =\frac{\sum_{0}^{t}\left(0.01-C\right)dt}{0.01t}\times 100\%$$

Here, η is the CO elimination efficiency/%, C is the real-time CO concentration (volume fraction), and t is the elimination time/s.

## 4. Conclusions

This study successfully developed a Cu(I)-based deep eutectic solvent (DES) system, [Emim]Cl-CuCl-1.0E, exhibiting low viscosity and high activity under ambient temperature and pressure. Comparative analysis with an ethylene glycol (EG)-modified system revealed that the introduction of ethanol significantly enhances the CO elimination performance. This improvement is primarily attributed to the lower viscosity of ethanol compared to ethylene glycol, which effectively reduces the mass transfer resistance of CO in the liquid phase, thereby increasing the reaction efficiency.

Regarding influencing factors, elevated temperature was found to lower the solvent viscosity and enhance gas diffusion, promoting the forward reaction; however, it concurrently led to a reduction in the system’s saturated adsorption capacity. The CO elimination performance showed a positive correlation with contact time, with the developed system achieving complete elimination of 1% CO within approximately 2.3 s. Stability tests indicated a gradual decline in performance with prolonged sealed storage, mainly associated with the ingress of atmospheric moisture and oxygen.

Mechanistic studies established that CO elimination primarily results from its coordination with Cu(I) ions in the system, forming a stable Cu(CO)^+^ complex. Raman spectroscopy analysis further elucidated that the addition of ethanol effectively increases the coordination number of Cu^+^, facilitating its binding with more ligand molecules. This process significantly enhances the activity of the Cu(I) ions and the overall CO elimination capacity of the system.

## Data Availability

The data presented in this study are available on request from the corresponding authors.
